# Tuning the Properties of Xylan/Chitosan-Based Films by Temperature and Citric Acid Crosslinking Agent

**DOI:** 10.3390/polym16172407

**Published:** 2024-08-24

**Authors:** Martina Camaño Erhardt, Yamil Nahún Solier, María Cristina Inalbon, Paulina Mocchiutti

**Affiliations:** 1 Instituto de Tecnología Celulósica, Facultad de Ingeniería Química, Universidad Nacional del Litoral, Santiago del Estero 2654, Santa Fe S3000AOJ, Argentina; martinacamano2@gmail.com (M.C.E.); ysolier@fiq.unl.edu.ar (Y.N.S.); cinalbon@fiq.unl.edu.ar (M.C.I.); 2 Consejo Nacional de Investigaciones Científicas y Técnicas (CONICET), Buenos Aires 1425, Argentina

**Keywords:** esterification, amidation, mechanical properties, thermal properties, water resistance

## Abstract

Petroleum-based food packaging causes environmental problems such as waste accumulation and microplastic generation. In this work, biobased films from stable polyelectrolyte complex suspensions (PECs) of xylan and chitosan (70 Xyl/30 Ch wt% mass ratio), at different concentrations of citric acid (CA) (0, 2.5, 5, 7.5 wt%), were prepared and characterized. Films were treated at two temperatures (135 °C, 155 °C) and times (30 min, 60 min) to promote covalent crosslinking. Esterification and amidation reactions were confirmed by Fourier Transform Infrared Spectroscopy and Confocal Raman Microscopy. Water resistance and dry and wet stress–strain results were markedly increased by thermal treatment, mainly at 155 °C. The presence of 5 wt% CA tended to increase dry and wet stress–strain values further, up to 88 MPa—10% (155 °C for 60 min), and 5.6 MPa—40% (155 °C for 30 min), respectively. The UV-blocking performance of the films was improved by all treatments, as was thermal stability (up to T_onset_: 230 °C). Contact angle values were between 73 and 84°, indicating partly wettable surfaces. Thus, thermal treatment at low CA concentrations represents a good alternative for improving the performance of Xyl/Ch films.

## 1. Introduction

At present, the development of new biobased and biodegradable materials for food packaging is a major challenge for researchers. The problem is due to conventional petroleum-based films that produce serious environmental impact such as waste disposal, generation of greenhouse gases, and pollution of oceans and seas. As consumer awareness of sustainability grows, companies are increasingly inclined to adopt environmentally friendly packaging practices. Zhang et al. [1] recently reported that around 40% of the plastic waste worldwide comes from food-packaging films. These statistics highlight the urgent need to explore sustainable alternatives for food packaging, among which polysaccharide-based films excel.

It has been shown that the combination of polysaccharides, at specific mass ratios and medium conditions, can be an alternative for the preparation of films with acceptable mechanical properties [2,3,4,5]. Polymers themselves, as well as the intermolecular physical interactions between polysaccharides, such as ionic and secondary interactions (hydrogen bonds and Van der Waals forces), are responsible for the formation of a strong network. Nevertheless, because physical interactions are reversibly linked [6], they weaken in the presence of water, mainly the hydrogen bond interactions [5]. Thus, films show poor resistance to water.

To reduce this effect, permanent networks can be obtained by the production of covalent crosslinking between the functional groups of polysaccharides, either in the absence or presence of a crosslinking agent.

Taking into account our background on xylan/chitosan films [3,7,8,9] and the results obtained recently by other authors using different compounds to covalently crosslink polysaccharides [1,10,11], it is interesting to evaluate the mode by which a simple thermal treatment, as well as the addition of citric acid followed by a thermal treatment, improves the general properties of xylan/chitosan films due to the formation of covalent crosslinking.

Glucuronoxylan, or just xylan (Xyl), is a hemicellulose present in hardwood trees, such as poplar wood, and represents 20–35% of the lignocellulosic material. It can be isolated from the rest of the matrix (cellulose, lignin, and extractives) by alkaline extraction [8]. Xylan is a weak anionic polyelectrolyte due to the presence of carboxylic acid groups in its chemical structure. On the other hand, chitosan (Ch) is a natural and linear polysaccharide, composed of β-(1,4)-N-acetyl-D-glucosamine units derived from the deacetylation of chitin present in shrimp, crustaceans, and fungal cell walls [12]. It is a weak cationic polyelectrolyte, and its number of amine groups depends on the degree of deacetylation. Thus, the combination of Xyl and Ch solutions at a specific mass ratio and pH allows the formation of stable cationic polyelectrolyte complex (PEC) suspensions. 

Crosslinking agents should contain at least two functional groups to react with polymer chains to form a three-dimensional network. Citric acid (CA) has multifunctional groups: three acid groups and one hydroxyl group (Graphical Abstract). At different pH values, the acid groups of citric acid can be ionized or non-ionized. While the ionized groups can form ionic crosslinks, the non-ionized groups, under high temperature, can form covalent crosslinks [1]. This compound is commonly employed in the food industry due to its high safety profile, non-toxic nature, and biodegradability. It is recognized as safe by the FDA (Food and Drug Administration, USA) and EFSA (European Food Safety Authority) [13]. Not only can CA act as a crosslinking agent but it can also contribute to the plasticity of the film. However, note that an increase in plasticity leads to an undesired increase in hydrophilicity [14]. 

It has been reported that at high temperatures and low pH values, several reactions, such as esterification and amidation, can occur when polysaccharides and citric acid are present. Esterification is the reaction between carboxylic acid groups (or one of their derivatives) and hydroxyl groups to produce esters [1,15] and amidation is the reaction between carboxylic acid groups and cationic amines (NH_3_^+^). According to McMurry [16] and Tasselli [17], imine formation can also occur between primary amine and aldehyde groups. Carbohydrate hydrolysis can also take place under acidic environmental conditions [1]. Thus, to obtain crosslinks avoiding hydrolysis, the amount of CA added and the conditions for crosslinking reactions should be optimized. 

Studies have shown crosslinking reactions between polymers containing different functional groups resulting from thermal treatment [18,19,20]. Nurkeeva et al. [18] reported that thermally induced intermacromolecular esterification reactions can occur between the carboxylic groups of polyacrylic acid (PAA) and the hydroxyl groups of polyvinyl alcohol (PVA). Blasques et al. [20] reported crosslinked reactions between xanthan chains via esterification reactions at 165 °C in the absence and presence of citric acid. Azeredo et al. [21] reported that citric acid can react with the hydroxyl groups of glucose to form covalent bonds between starch molecules, enhancing the performance of starch films. In addition, it has been reported that sodium hypophosphite can act as a catalyst, accelerating the esterification of polycarboxylic acids by increasing the formation rate of cyclic anhydride intermediate in the CA molecule [10].

In this work, Xyl/Ch polyelectrolyte complex suspensions were prepared containing citric acid at different concentrations (0, 2.5, 5, and 7.5 wt% on a dry mass basis and sodium hypophosphite as the catalyst). Suspensions were characterized by determining zeta-potential and average particle size. Subsequently, films were formed using the casting/evaporation technique. The films were thermally treated (135 °C and 155 °C) for 30 min and 60 min, and the chemical modifications were analyzed by Fourier Transform Infrared Spectroscopy (FTIR) and Confocal Raman Microscopy (CRM). At high temperatures, esterification crosslinking reactions are expected to occur between the polymeric chains of xylan–chitosan and xylan–xylan, or between citric acid–xylan and citric acid–chitosan, while amidation reactions are expected to take place between xylan–chitosan and citric acid–chitosan. Conversely, imine reactions could occur between chitosan and terminal carbonyl groups of xylan. 

To the best of our knowledge, this is the first study on the influence of thermal treatment and citric acid addition on the crosslinking of Xyl/Ch films. Dry and wet mechanical properties and swelling capacity were also evaluated. Film morphology was characterized via SEM analysis. Contact angle measurements were performed to assess the surface hydrophobicity of films. Finally, the thermal stability of the films was analyzed by thermogravimetric analysis (TGA).

## 2. Materials and Methods

### 2.1. Xylan 

Xylan was extracted from twelve-year-old poplar wood by alkaline treatment and bleached using hydrogen peroxide (10% *w*/*w*), as previously explained [8]. Its molecular weight was 56.3 kDa (polydispersity index 1.50), and its glucuronic acid group content was 0.63 meq/g. For film formation, a 4 g/L xylan solution was prepared by dissolving it in distilled water and heating it in a boiling water bath for 15 min.

### 2.2. Chitosan

Chitosan (Ch) was supplied by Sigma-Aldrich (Darmstadt, Germany). The viscosity average molecular mass was 270 kDa, and the degree of deacetylation weight was 78.1% [7]. A 2.5 g/L solution was prepared by the slow addition of 0.25 g/L acetic acid solution to the Ch powder, maintaining continuous mixing to achieve a homogeneous solution. The preparation was magnetically stirred for 24 h, and filtered with a porous glass filter. The pH of the solution was 4.0.

### 2.3. Citric Acid and Sodium Hypophosphite

Citric acid and sodium hypophosphite (analytical grade powders) were supplied by Sigma-Aldrich (Darmstadt, Germany) and used to prepare 10 g/L and 5 g/L solutions, respectively.

### 2.4. Preparation of Xyl/Ch PEC Suspensions in the Presence of Citric Acid and Sodium Hypophosphite

Polyelectrolyte complex (PEC) suspensions (3.55 g/L) were prepared by combining xylan (Xyl) and chitosan (Ch) at pH 4.0. This pH value was selected to achieve the esterification reaction. A Xyl/Ch mass ratio of 70/30 wt% was used for the study, according to previous results [3]. PEC suspensions were prepared in the presence of different concentrations of citric acid as a crosslinking agent (0, 2.5, 5.0, and 7.5 wt% relative to the total dried mass) and sodium hypophosphite as a catalyst (adding 50% of the acid amount: 0, 1.25, 2.5, and 3.75% relative to the total dried mass, respectively, as reported by Zoldners and Kiseleva [22]). 

As preliminary trials, PECs containing 10 wt% CA were also prepared. Nevertheless, this high concentration of CA led to the formation of sizeable aggregates, resulting in the formation of non-uniform films with poor mechanical properties. 

Citric acid and sodium hypophosphite solutions were added to the xylan solution, and the pH was adjusted. The resulting solution was then added to the chitosan solution at 90 mL/h using a syringe pump. The suspension obtained was later homogenized using an ultrasonic homogenizer (2 min, 500 W, 40% amplitude).

### 2.5. Determination of Particle Size and Z-Potential Values of PECs

PEC size and z-potential value were estimated using Zetasizer Nano ZS90 (Malvern Instruments, Worcestershire, UK). PEC suspensions were prepared at a lower concentration (1.55 g/L in water at pH 4.0) to be measured in the equipment. At least eight measurements were made for each condition at 25 °C. The refractive index of water (1.334) [23] and a viscosity of 0.8872 mPa·s were used to calculate the properties.

### 2.6. Preparation of Xyl/Ch Films and Crosslinking

PEC suspensions were cast on silicone molds, and the solvent was evaporated in an air-forced oven at 40 °C until dry. Films were removed from the molds and conditioned for at least 2 h at 50% relative humidity (RH) and 23 °C. Film treatment was conducted as follows: (1) Films were dried in a vacuum oven at 60 °C for 48 h to remove the equilibrium moisture content. According to Wing [24], pre-drying films at a low temperature reduces excess hydrolysis during crosslinking. (2) To induce crosslinking, the pre-dried films were placed between two steel plates (using thin silicone sheets between the film and the plates), and a weight mass exerting a pressure of 30 kgf/m^2^ was placed on top to avoid film deformation during treatment. Afterwards, the system was placed in an oven (30 and 60 min) at different temperatures, 135 °C and 155 °C. A high polysaccharide content and a sufficiently high temperature are required to evaporate the water produced in the reaction to be considered irreversible [25]. Untreated films were used as a reference. 

### 2.7. Washing Step

After crosslinking treatment, films were conditioned at 50% RH and 23 °C for 10 h, and then thoroughly washed for 60 min. Each film was placed between two meshes (for protection), immersed in water at pH 4.0, and magnetically stirred. This part of the procedure represents an important step for the removal of salts, the unreacted CA, and the catalyst. Subsequently, the films were dried under tension (pressure of 100 kgf/m^2^), as explained in [26], and stored at 50% RH and 23 °C for further characterization.

### 2.8. FTIR Spectroscopy 

The interaction between film components was determined by FTIR analysis. Film samples were reduced to their smallest possible size using scissors and placed under vacuum at 60 °C for 48 h. Potassium bromide was added to the samples and pressed to form pellets for later use. FTIR spectra were obtained using a Fourier transform spectrophotometer (Shimadzu FTIR-8000 spectrometer, (Shimadzu Co., Kyoto, Japan)) and recorded in the 700–4000 cm^−1^ wavelength range with a resolution of 8 cm^−1^. Each sample was scanned 40 times. Using data analysis and graphics software, the deconvolutions of the interest regions were made to distinguish specific functional groups. 

### 2.9. Confocal Raman Microscopy

Confocal Raman Microscopy was also used to analyze the functional groups in the films treated at 155 °C. The equipment used was a Renishaw Confocal Raman Microscope (Renishaw, Gloucestershire, England), featuring 2 laser excitation lines at 514 and 785 nm. Three scans of 10 s each were conducted for each spectrum. A 785 nm laser at 100% power and a 100× objective were employed. For comparison, the spectra were normalized.

### 2.10. UV-vis Light Transmittance 

The behavior of the films exposed to UV-visible light was studied using a spectrophotometer Cecil 3055 (Cecil Instruments Limited, Cambridge, England) in the range 200 to 800 nm [27]. Films were cut into 12 × 30 mm pieces and placed on the surface of an empty quartz cuvette. The empty cell was used as the blank.

Opacity was measured at 600 nm and calculated with Equation (1), as in [27].

(1) $$Opacity\;value=\frac{{Abs}_{600}}{t}$$

where *Abs*_600_ is the absorbance at 600 nm and *t* is the film thickness (mm).

### 2.11. Mechanical Film Properties 

Dry and wet tensile strengths and elongations at break were measured using an INSTRON 3340 universal testing machine (Instron Corporation, Norwood, MA, USA) with a 1000 N load cell at 23 °C and 50% RH. The trials were conducted following the American Society for Testing and Materials (ASTM D882) standard method [28] with certain modifications: rectangular specimens of 30 mm × 5 mm were prepared, the speed was 3 mm/min, and the initial grip distance was 22 mm. Wet tensile strength was determined after film immersion in distilled water at 23 °C for 1 h, following the International Organization for Standardization ISO 3781:1983 standard method [29]. For each condition, no fewer than ten specimens were used for both dry and wet determinations.

### 2.12. Swelling Behavior, Solubility, and Equilibrium Moisture Content

Conditioned films were cut into squares of approximately 1 cm^2^ each. Then, they were placed in a vacuum oven at 60 °C for 48 h, and the initial dry weight of each sample was recorded (*Weight_dry initial_*). The samples were immersed in distilled water for 4 h. Subsequently, the excess liquid was removed by pressing the samples between two blotters for the weight to be recorded again (*Weight_wet final_*). Finally, the samples were placed in vacuum under the same conditions and then weighted (*Weight_dry final_*). The swelling capacity and solubility were calculated according to Equations (2) and (3), respectively:

(2) $$Swelling\;(\%)=\frac{{Weight}_{wet\;final}-{Weight}_{dry\;final}}{{Weight}_{dry\;final}}\times 100$$

(3) $$Solubility\left(\%\right)=\frac{{Weight}_{dry\;initial}-{Weight}_{dry\;final}}{{Weight}_{dry\;initial}}\times 100$$

### 2.13. Thermal Gravimetric Analysis 

The thermal stability of films was evaluated by thermogravimetric analysis (TGA). The equipment used was a TGA/SDTA851e Module—Mettler Toledo (TA Instruments, model Q500, New Castle, DE, USA). The films (200 mg) were initially conditioned at 23 °C and 50% RH for 24 h, and subsequently heated from room temperature to 800 °C at a heating rate of 10 °C/min while being exposed to a nitrogen stream. Thermogravimetric curves were built, and the onset (T_onset_) and endset (T_endset_) temperatures were determined, as they correspond to temperatures at which significant loss of mass begins and ends, respectively. In addition, the degradation temperature (T_deg_) obtained using the maximum of the derivative of the TG curve; and the percentage mass residue evaluated at 800 °C (W_residual_) were determined from the curves.

### 2.14. Scanning Electron Microscopy (SEM)

The surface morphology of films was examined using Scanning Electron Microscopy with Energy-Dispersive Spectroscopy (SEM-EDS, Zeiss Instrument, model CrossBeam 350, Carl Zeiss Microscopy GmbH, Jena, Alemania) in the secondary electron imaging mode using an accelerating voltage of 1 kV. Samples were prepared according to the method described by Schnell et al. [7]. The films were coated with an approximately 10 nm thick gold layer before evaluation.

### 2.15. Contact Angle 

The hydrophobicity/wettability of the film surface was determined by measuring the static contact angle of water on the film surface. Ten distilled water droplets (approximately 18 μL) were deposited onto the dried film surfaces. Contact angles were processed using the LBADSA method implemented in ImageJ 1 software [30].

### 2.16. Statistical Analysis 

The thermal treatments of the films for different time spans and different citric acid concentrations were considered independent experiments. Results were analyzed by one-way analysis of variance (ANOVA), using the RStudio program (version 4.2.3). Differences between the mean values of the film properties were determined using Tukey’s test. The significance of the difference was determined at a 95% confidence level (α = 0.05). The results are presented as the mean ± standard deviation (SD), and *p*-values are reported.

## 3. Results

### 3.1. Z-Potential and Average Particle Size 

Table 1 shows the z-potential values, average particle sizes, and polydispersity index (PDI) of the Xyl/Ch PEC suspensions. Taking into account Xyl/Ch mass ratio and CA content, the total charge of the PECs was also estimated for each condition (Table 1). For the estimation at pH 4.0: chitosan was completely ionized (75% deacetylated) and xylan (0.1 eq/monomer) was 60% ionized [31]. Also, knowing the dissociation constants of CA (pKa values: 3.13, 4.76, and 6.4 [32]), it was expected that, at pH 4.0, the two acidic groups were partially ionized, while the other acidic group and hydroxyl group remaineed mostly non-ionized (Graphical Abstract). Thus, 1 eq/mol was considered for citric acid. The table shows that all z-potential values were higher than 30 mV, indicating that the suspensions were stable, although the addition of CA gradually reduced the value (from 37 to 30 mV when 7.5 wt% CA was used, *p* = 0.00). It was probable that the anionic groups of the citric acid would crosslink with the free cationic NH_3_^+^ chitosan groups (ionic crosslink), reducing the z-potential as the total cationic charges of the PECs. However, the system remained cationic and relatively stable.

Additionally, the non-ionized acid groups of the CA and the hydroxyl groups could form hydrogen bonds with polysaccharides. Both interactions significantly reduced the average particle size from 1078 to 876 nm when 7.5 wt% CA was used (*p* = 0.00). The PDI value obtained suggests midrange polydispersity suspensions.

Note that due to the increase in concentration during the evaporation of the PEC suspension for film formation, the stability of the suspensions was visually reduced, particularly when CA concentration reached higher values. 

### 3.2. FTIR Analysis

**Figure 1:**
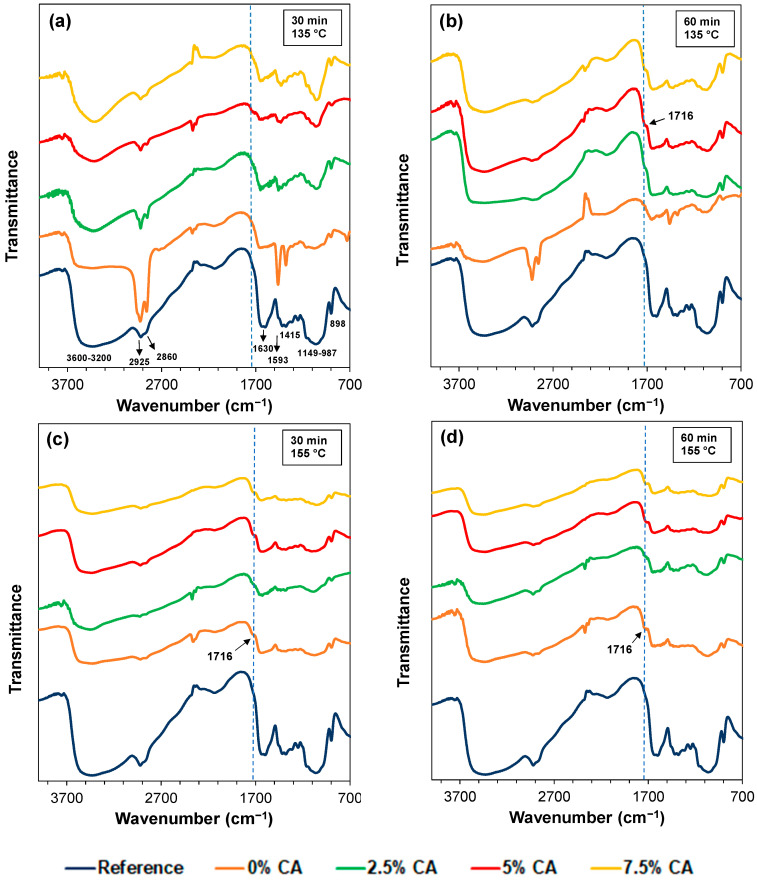
FTIR spectra of the films thermally treated at 135 °C for 30 min (**a**) and 60 min (**b**), and at 155 °C for 30 min (**c**) and 60 min (**d**) at different CA concentrations. The spectrum of the reference film is shown in all figures to allow the comparison.

Figure 1 shows the FTIR spectra of the reference film and films containing citric acid thermally treated at 135 °C and 155 °C in the 4000–700 cm^−1^ range.

The peaks in the spectrum of the Xyl/Ch reference film are the same as those in our previously reported work [7], detailed in Figure 1a. A broad band can be observed between 3600 and 3200 cm^−1^, which is due to the overlaying of the stretching vibration band of the xylan hydroxyl groups (O–H) and the stretching vibration band of the primary amines (N–H) and secondary amides of chitosan. This broad band is also due to hydrogen bonds [33]. The spectrum also shows asymmetric and symmetric aliphatic C–H stretching vibration peaks of CH_3_ groups at 2925 cm^−1^ and 2860 cm^−1^, respectively. The characteristic peaks of chitosan are attributed to amide-stretching vibration (C=O) at around 1630 cm^−1^, and at around 1593 cm^−1^, to N–H deformation of primary amines overlapping amide II vibration (1570–1520 cm^−1^). These peaks are also shown as broad bands [33]. This region also exhibits the vibrations of the asymmetric deformation of NH_3_^+^ amine salts (1625–1560 cm^−1^) [33], as well as the stretching vibration of ionized carboxylic groups (1610–1550 cm^−1^) of xylan [34,35]. Due to overlap, it is difficult to detect modifications such as those of amidation and imine reactions. The characteristic vibrations of the groups present in polysaccharides are also observed (–CH_2_ bending vibration at 1415 cm^−1^ and–CH_3_ symmetrical deformation at 1380 cm^−1^, overlapping with O–H in-plane deformation at 1440–1390 cm^−1^). A peak can be observed at around 1250 cm^−1^ due to the N–H deformation of secondary amides (amide III band). In addition, a band can be observed between 1170 and 987 cm^−1^, where several vibrations occur: 1150 cm^−1^ asymmetric stretching of the C–O–C bridge, 1126 cm^−1^ and 1018 cm^−1^ skeletal vibration due to C–O stretching of secondary alcohols found in chitosan and xylan that overlap with 1041 cm^−1^, C–O bond-stretching of primary alcohols in chitosan [34,36]. The vibration of β-glycosidic linkages between monomers can be observed at 898 cm^−1^ [37]. On the other hand, a peak at around 2400–2300 cm^−1^, assigned to the vibration of CO_2_ in the environment, is observed in all the spectra. The peak may appear upwards or downwards, depending on whether CO_2_ absorption in the sample is higher or lower than that in the background.

Figure 1a,b also show the spectra of films treated at 135 °C for 30 and 60 min, respectively. Treatment for 30 min showed no changes in the FTIR spectra when 0 wt% and 2.5 wt% CA were used. However, using 5 wt% and 7.5 wt% CA, a new peak at around 1716 cm^−1^ was observed. When the treatment was for 60 min, this peak appeared for all the cases containing CA. This peak can be attributed to a C=O ester vibrational-stretching band [38,39,40,41], indicating the occurrence of esterification reactions. Nevertheless, in the 1730–1710 cm^−1^ region, non-ionized carboxylic acid groups ν(C=O)_COOH_ could also vibrate [34]. Deconvolution of the band in the 1750–1620 cm^−1^ region was made to distinguish carboxylic and ester groups for quantitative evaluation. It was found that, when thermal treatment at 135 °C—30 min was applied on films containing 5 wt% and 7.5 wt% CA, the percentage contribution of ester groups to the total area of the band was 5% and 2%, respectively. On the other hand, when 135 °C—60 min was applied, the contribution of ester groups was 15%, 15%, and 24%, when 2.5 wt%, 5 wt%, and 7.5 wt% CA were used, respectively. 

The FTIR spectrum of citric acid powder (Figure S1) reveals two characteristic peaks of similar intensities at 1747 cm^−1^ and 1697 cm^−1^. Since the peak at 1747 cm^−1^ was not observed in the spectra of all the evaluated films, it can be confirmed that the washing step was effective enough to remove the unreacted citric acid. Conversely, the mono-esters and di-esters resulting from esterification reactions between citric acid and polymers remained in the films anchored to the polysaccharides. These reactions exposed non-ionized and ionized carboxylic acid groups to the solution (Graphical Abstract). 

Additionally, Figure 1c,d show the FTIR spectra of films treated at 155 °C for 30 and 60 min, respectively. Both figures already show the peak at 1716 cm^−1^ after the thermal treatment and use of CA, indicating that esterification reactions had indeed occurred. Deconvolution of the band in the 1750–1620 cm^−1^ region was also performed. When thermal treatment—without CA—was applied, the contribution of ester groups to the total area of the band was 11% and 12% for 30 and 60 min, respectively. When CA was used, values increased to 18% and 20% for 5 wt% and 7.5 wt% CA, respectively, for both times studied.

Hence, when 135 °C was used, 5 wt% CA—30 min and 2.5 wt% CA—60 min were enough to generate this crosslinking. On the other hand, at 155 °C, a simple thermal treatment was sufficient to produce ester groups, and the use of CA increased this interaction.

### 3.3. Mechanical Properties

#### 3.3.1. Dry State

During thermal treatment, esterification and amidation reactions, imine formation, and hydrolysis of carbohydrates occur. The displacement of these reactions to reaction products depends on the conditions of the medium (CA concentration, time, temperature, and pH). More fragile films can be produced by the hydrolysis of carbohydrates, while crosslinking reactions can be favored at high temperatures, thus enhancing film mechanical properties. 

Menzel et al. [11] used citric acid for crosslinking starch films and reported that at low pH (1.9, 2.5), the hydrolysis of starch also occurs. In our case, films were prepared at pH 4.0 because, at lower pH values, we found that such films were burned during thermal treatment.

**Figure 2:**
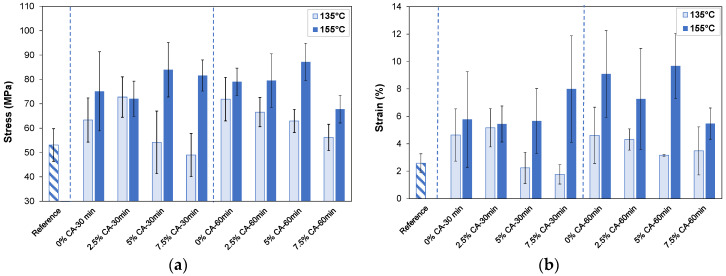
Dry tensile stress (**a**) and dry strain at break (**b**) of reference film (striped pattern), as well as thermally treated films (135 °C, 155 °C) for two times (30 min, 60 min) at different concentrations of CA (0 wt%, 2.5 wt%, 5 wt%, 7.5 wt% CA). Blue dashed lines separate different treatment times.

Figure 2a shows the tensile strength of the films after different treatments. Compared with the reference film (53 MPa), when the exclusive thermal treatment was used at 135 °C, it was necessary to extend the time to 60 min to significantly improve this property to 72 MPa (*p* = 0.00). On the other hand, thermal treatment at 155 °C increased this property to 75 MPa for 30 min (*p* = 0.00), and to 79 MPa for 60 min (*p* = 0.00). 

When CA was added, different behaviors were observed at the two temperatures evaluated. At 135 °C for 30 and 60 min, the films containing citric acid (7.5 wt% CA) showed lower tensile strength compared to that of their respective films which had only been thermally treated (0% CA—30 min (*p* = 0.008) and 0% CA—60 min (*p* = 0.005)). This reduction can be attributed to the effect of the hydrolysis reaction, which was dominant over the crosslinking reaction. No significant difference was observed when 2.5 wt% and 5 wt% CA were used (*p* > 0.05). On the other hand, at 155 °C and independent of time (30 or 60 min), the addition of 5 wt% CA showed a tendency to increase the tensile stress of the thermally treated films (0% CA—30 min and 0% CA—60 min) by 12% and 10%, respectively, although these increases were not statistically different at the 95% confidence level (*p* > 0.05). Particularly, the maximum value was reached when 5 wt% CA—60 min was used (stress at break of 87 MPa). In this case, crosslinking reactions were favored over carbohydrate hydrolysis, improving the strength of films. Higher citric acid concentrations (7.5 wt% CA) reduced this property compared with films containing 5 wt% CA when treatment was performed for 60 min (*p* = 0.003).

Figure 2b shows that the strain at break was significantly increased by thermal treatments, especially when performed at 155 °C for 60 min (*p* = 0.00). When tensile strength and elongation at break improve together, a polymer network with higher homogeneity is obtained [7,14]. With citric acid in the film, different behaviors were observed when thermal treatment was applied at 135 °C or 155 °C. At 135 °C for 30 min, citric acid significantly reduced strain when 7.5 wt% CA was used (*p* = 0.00), making the film more rigid. At 155 °C, no significant differences were observed when CA was used, compared with 0% CA—30 min and 0% CA—60 min (*p* > 0.05). Different authors [1,42] reported that excess CA can increase film strain. In our case, as films were thoroughly washed and unreacted CA was removed, elongations were not expected to be significantly modified. 

It can be concluded that the film containing 5 wt% CA thermally treated at 155 °C for 60 min showed the best dry stress–strain mechanical properties. The contribution of thermal treatment to these values was markedly high. 

Other authors have shown a significant increase in the tensile strength, using CA as crosslinking agent, of biobased polysaccharides films. Particularly, Wang et al. [14], showed that xylan/poly(vinyl alcohol) (25/75 wt%) films containing 10 wt% CA and treated at 110 °C for 2.5 h increased their tensile strength from 30.3 MPa to 49.3 MPa and decreased strain from 10.7% to 6.8%. Using corn starch-based films, it was shown that the addition of 5 wt% CA and 2.5% sodium hypophosphite, followed by film thermal treatment at 130 °C for 30 min, increased the tensile strength from 10.8 to 18.2 MPa and the elongation from 3.15% to 4.2% [41]. Furthermore, Wu et al. [40], demonstrated that 10 wt% and 15 wt% CA increased the tensile strength of potato starch/chitosan film from 9.7 to 12.5 MPa. In this last case, only ionic crosslinking between the agent and the polysaccharides is expected. The CA crosslinking agent, as well as the catalyst, can also be used for improving the mechanical properties of protein-based films [43]. Thus, the tensile strengths of the films obtained in this work are comparable to those reported in the literature, suggesting good potential for their development as packaging.

The elastic modulus of the reference film was 3500 MPa. Neither thermal treatment nor CA treatment significantly modified this property, which was around 3000–4100 MPa. 

#### 3.3.2. Wet State 

**Figure 3:**
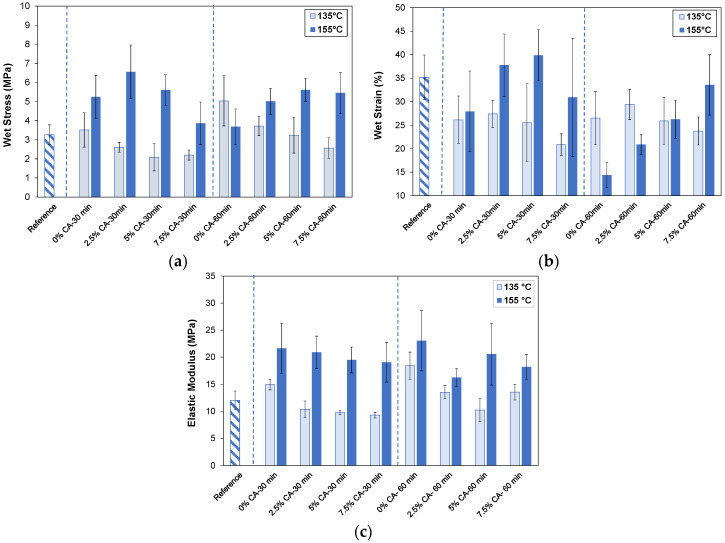
Wet mechanical properties obtained after immersion of films in water for 1 h. Wet tensile stress (**a**), wet strain at break (**b**), and elastic modulus (**c**) of reference film (striped pattern), and thermally treated films (135 °C, 155 °C) for two times (30 min, 60 min) at different concentrations of citric acid (0 wt%, 2.5 wt%, 5 wt%, 7.5 wt% CA). Blue dashed lines separate different treatment times.

Figure 3a shows the results of the wet tensile strength. All values were lower than those of the dry tensile strength, indicating that water acted as a plasticizer, and that the structural conformation of the film was modified. It is well known that a plasticizer increases interstitial volume and polymer mobility, resulting in a less dense polymeric network with weaker intermolecular forces [14]. In this case, hydrogen bonds are expected to weaken in the presence of water, while covalent and other physical crosslinks remain present. Chen et al. [5] compared the mechanical properties of dry chitosan/CMC films to those of hydrated films. They reported that hydrogen bonds were disrupted by water molecules; yet, due to some physical crosslinking points resulting from polyelectrolyte complexation, their integrity and dimensional stability were maintained, as also found in our work. 

Particularly, the figure shows that thermal treatment at 135 °C had a favorable effect on stress when applied for 60 min (*p* = 0.00). Nevertheless, when CA was used, this property decreased in all cases, except when 2.5 wt% CA was used for 30 min (*p* = 0.15). As previously explained for dry mechanical properties, hydrolysis of carbohydrates in the acid medium occurred, weakening the film. The figure also shows that the application of thermal treatment at 155 °C for 30 min was sufficient to increase the tensile strength of the reference film (*p* = 0.00). On the other hand, at 155 °C for 60 min with 2.5 wt%, 5 wt%, and 7.5 wt% CA, tensile strength significantly increased (*p* = 0.038, *p* = 0.0002, and *p* = 0.001, respectively) compared with 0% CA—60 min, suggesting that certain physical crosslinks between xylan and chitosan, and the covalent crosslinks produced by the reactions, improved the polymeric network. 

Figure 3b shows that strain at break also increased when 5 wt% CA and 155 °C were used (*p* = 0.006), compared to 0 wt% CA—30 min. When only thermal treatment was performed for 60 min, the strain at break decreased significantly (*p* = 0.00). However, 5 wt% and 7.5 wt% CA allowed for the flexibility of these last films to be slightly recovered (*p*= 0.003 and *p* = 0.00, respectively).

It can be concluded that the best wet mechanical properties (tensile strength and strain at break) were obtained when films containing between 2.5 and 5% CA were thermally treated at 155 °C for 30 min. 

Similar results were obtained by Liu et al. [44] and Li et al. [45]. Particularly, Liu et al. [44] prepared films using chemically modified soy protein, cured for different times (2–20 min) and temperatures (140–180 °C). Their wet tensile strengths were between 3 and 7 MPa (films conditioned at 90% RH, 48 h). Additionally, Li et al. [45], prepared films using hemicellulose crosslinked with citric acid, and showed wet tensile strengths between 4 and 8 MPa (films conditioned at 95% RH, 24 h). The authors suggested that films with these wet tensile strength values were stable in water and could be used for applications such as packaging for wet food. 

Figure 3c shows that the elastic modulus of the reference film is 12 MPa. Although thermal treatments (135 °C and 155 °C), independent of time, significantly increased this property in all cases (*p* <0.05), CA tended to reduce the elastic modulus. The values obtained after thermal treatment at 155 °C were higher than those at 135 °C, thus intensifying film rigidity due to crosslinking.

### 3.4. Swelling and Solubility 

**Figure 4:**
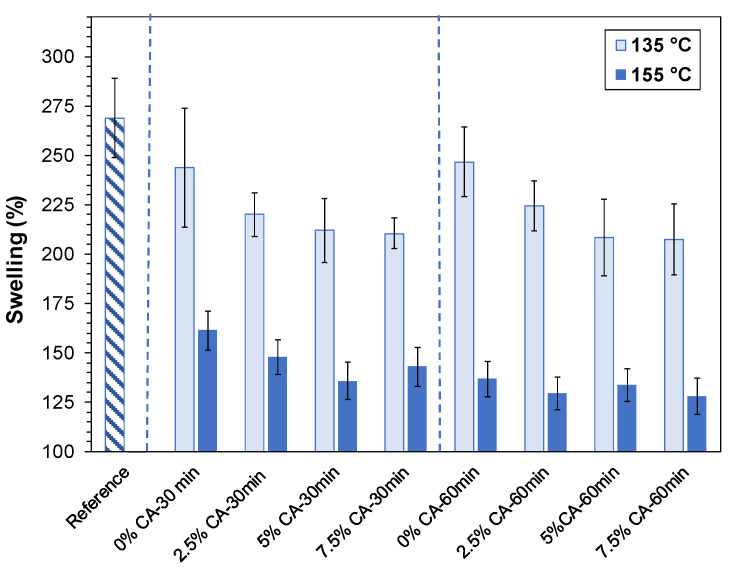
Swelling of the reference film (striped pattern), as well as thermally treated films (135 °C, 155 °C) for two times (30 min, 60 min) at different concentrations of CA (0 wt%, 2.5 wt%, 5 wt%, 7.5 wt% CA). Blue dashed lines separate different treatment times.

Swelling is related to the quantity of water absorbed by a film. Low values are required for food packaging applications, as they indicate high resistance to water. Figure 4 shows the swelling capacity of films after immersion in water for 4 h. Not only did all the films maintain their integrity, but they also swelled and resisted dissolution. Crosslinking reactions can enhance the water resistance of the material by chemically and physically reinforcing the network [11,40], which is one of the main objectives of this work. The figure shows that the swelling capacity of the reference film is 269%. Thermal treatment at 135 °C for 30 min and 60 min slightly reduced this property to 244% and 249%, respectively, although it was not statistically significant (*p* = 0.27 and *p* = 0.40, respectively). It was necessary to add 7.5 wt% CA and thermally treat the films at 135 °C for 60 min to significantly reduce the swelling capacity of the film to 207% (*p* = 0.028). 

At 155 °C, the behavior was markedly different. Thermal treatment without citric acid was significantly effective, decreasing swelling by 39% at 30 min (*p* = 0.00) and 49% at 60 min (*p* = 0.00) to 161% and 137%, respectively. Tukey’s test also showed that the difference between them was significant (*p* = 0.00). The occurrence of crosslinking reactions between xylan and chitosan was also confirmed by a decrease in swelling capacity and increase in water resistance. The presence of the crosslinking agent also reduced the swelling capacity of the film. At 155 °C for 30 min, addition of 5 wt% CA reduced the swelling capacity by 16%, compared to the same thermal treatment with no citric acid (*p* = 0.00), to 136%, in agreement with the improvement seen in wet tensile strength (Figure 3a). Several authors have also shown that the swelling capacity of biobased films decreased as esterification and crosslinking degrees increased [14,46]. 

No significant differences were observed at higher concentrations of CA. This phenomenon may be attributed to the excess unreacted carboxylic acid groups of the citric acid molecule anchored to the carbohydrates. Lipatova and Yusova [41] prepared starch films crosslinked with citric acid at high temperature. Using complexometric titration, they concluded that esterification reactions produced mono-, di-, and tri-esters of CA. Conversely, it has been reported that the probability of tri-esters is uncertain [11]. It is plausible that the favorable effect of reduced swelling due to intermolecular covalent bonds may be offset by the presence of exposed carboxylic acid groups, as well as OH groups, contributing to increased swelling.

The low swelling value obtained at 155 °C—60 min (137 g of water/100 g of film) could not be significantly reduced with citric acid (*p* > 0.05).

The solubility values of all the films were lower than 3% with no observable trend related to treatment conditions or CA content.

### 3.5. Characterization of Films Thermally Treated at 155 °C

Taking into account the results of the FTIR spectra, mechanical properties, and swelling capacity, it is necessary to reach a temperature of 155 °C to markedly improve film properties. Thus, films treated at 155 °C at different times and CA concentrations were characterized by Confocal Raman Microscopy, their microstructure, surface wettability, UV-visible light barrier, and thermal behavior. Results are shown in the following Section 3.5.1, Section 3.5.2, Section 3.5.3, Section 3.5.4 and Section 3.5.5.

#### 3.5.1. Confocal Raman Microscopy Analysis

**Figure 5:**
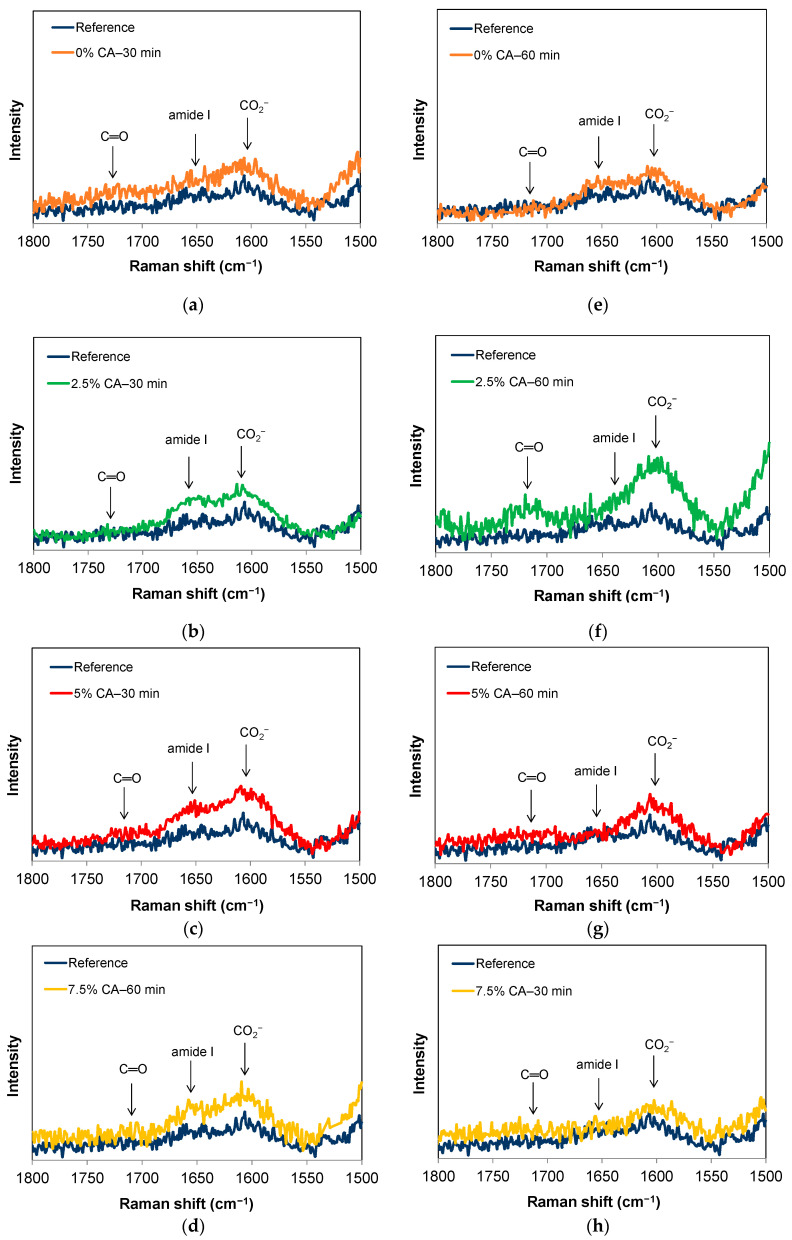
Confocal Raman Microscopy normalized spectra of the reference film and films treated at 155 °C for 30 min (**a**–**d**) and 60 min (**e**–**h**) at different concentrations of citric acid (0 wt%, 2.5 wt%, 5 wt%, 7.5 wt% CA). The spectrum for the reference film is shown in all figures to allow comparison.

Infrared and Raman spectroscopic methods are complementary for the analysis of chemical structures. Figure S2 shows the Raman spectrum of the reference film in the range of 3600–400 cm^−1^. Between 3580 and 3350 cm^−1^, the O–H stretching vibration of alcohols present in carbohydrates overlaps with the O–H stretching vibration due to hydrogen bonds. In addition, in the region of 3450–3180 cm^−1^, the stretching vibration of the N–H bond of primary amines NH_2_ and secondary amides of chitosan (hydrogen bonded) also vibrates [34]. At around 2900 cm^−1^, a sharp peak can be observed due to the symmetric stretching vibrations of –CH_3_ and –OCH_3_ from chitosan and xylan, respectively. The region of the 1800–1500 cm^−1^ spectra was analyzed in more detail (Figure 5). Figure S2 also shows a medium peak at 1467 cm^−1^ due to the deformation vibration (scissor) of –CH_2_, and at 1422 cm^−1^ and 1388 cm^−1^ due to the asymmetric and symmetric vibration bands of the methyl group, respectively. Moreover, –CH_2_ deformation vibration (wagging) can also be seen at 1322 cm^−1^. In the 1330–1215 cm^−1^ range, a band can also be observed due to the new amide III vibration [34]. Furthermore, a sharp peak at 1096 cm^−1^ can be observed due to the C–N stretching of amides [34]. The C–O–C vibration of carbohydrates at 902 cm^−1^ can also be identified, as well as the C–O deformation vibration at 494 cm^−1^ [34].

Figure 5 shows the Raman spectra in the region of 1800–1500 cm^−1^ where the functional groups of the reaction products, such as those from esterification and amidation, can be identified. Figure 5a–d, on the left side, show those films thermally treated for 30 min, while Figure 5e–h, on the right side, show the spectra corresponding to 60 min of treatment. Weak bands in the range of 1750–1725 cm^−1^ can be assigned to the C=O stretching vibration of ester groups that can partially overlap with the weak C=O stretching vibration of non-ionized carboxylic groups (1735–1700 cm^−1^) [34]. In addition, in the region between 1650 and 1550 cm^−1^, bands can be assigned to the vibration of amide I groups that can only partially overlap with the C=O asymmetric stretching vibration of ionized carboxylic groups (around 1600 cm^−1^). Figure 5a shows that thermal treatment for 30 min at 155 °C barely increased the intensity of the bands corresponding to the ester groups, amides, and ionized carboxylic acids, indicating that chemical reactions occurred. The areas of the bands in the region of 1670–1620 cm^−1^ associated with the amide I group’s vibrations were estimated. Compared with 0% CA—30 min, when 2.5 wt% CA was used (Figure 5b), a large increase was observed in this region (the area increased by 20%). Yet, when 5 wt% CA was used, the amide band increased even more (the area increased by 37% compared with 0% CA—30 min). The amide groups were more easily detected using this technique than via FTIR analysis. Bands attributed to esters and ionized carboxylic groups also increased when 5 wt% CA was used. These results agree with those obtained from FTIR analysis and confirm that citric acid was anchored through esterification reactions, producing crosslinks between polymers, as well as mono- and di- esters and amidation reactions. The intensities of the bands of the film containing 7.5 wt% CA thermally treated for 30 min (Figure 5d) were only slightly higher than the reference film.

When the films containing 5 wt% CA were thermally treated for 60 min, the intensities of the band of the amide I groups decreased (the area was reduced by 39% compared with 0% CA—60 min). On the other hand, the spectra of the film with 2.5% CA—60 min showed the most intense peak of esters and ionized carboxylic acids (the areas were increased by 150% and 36%, respectively). The ester peak was difficult to observe in the FTIR spectra for the last condition (Figure 1d). 

Vandenabeele et al. [47] also reported the vibration of C=O of esters at around 1710 cm^−1^ and C=O of amide I at around 1650 cm^−1^ when Raman spectroscopy was used for proteins. In addition, the appearance of a weak asymmetric stretching vibration at 1605 cm^−1^ was attributed to the presence of ionized carboxylic groups [35,48].

#### 3.5.2. UV Barrier and Opacity Properties

**Figure 6:**
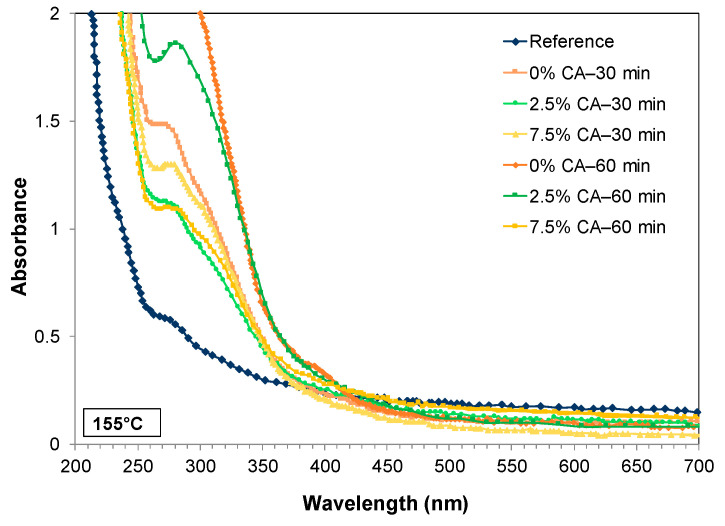
Absorbance spectra as a function of wavelength of the different films. Except for the reference film, all other films were treated at 155 °C.

It is well known that UV-light (200–400 nm) activates oxidation reactions in fatty and polyunsaturated food and can modify their organoleptic characteristics. Figure 6 shows the UV-visible absorbance spectra of the films (values also consider the light dispersed). Film thickness was 45–55 µm. All spectra showed saturation of absorbance between 200 and 230 nm. Compared to the reference film, the absorbance of the treated films was markedly higher, up to 350 nm, indicating better UV-barrier properties. The peak at 280 nm shows that thermal treatment increased the absorbance value, while the presence of citric acid slightly reduced this effect. Nevertheless, absorbance values were always higher than those of the reference film.

Table S1 shows the opacity values of the films (measured at 600 nm). Compared to the reference film (opacity 3.5), thermal treatment and CA reduced opacity (values around 2), probably due to crosslinking, producing a denser film with less light dispersion. Several authors [27,40,41,49] produced highly transparent films with opacity values lower than 2. Thus, it suggests that treatments improved film transparency. This feature is of major importance since the food contained in the packaging is clearly visible to the consumer [27].

#### 3.5.3. SEM Microscopy Analysis 

Film microstructure was analyzed using SEM. Figure S3 shows the surface of the reference film, as well as the films thermally treated (155 °C) or thermally treated in the presence of the crosslinking agent. All films showed a smooth and continuous structure, without cracks or pores, indicating that components were homogeneously mixed in the matrices. In our previous works [7,31] we had found that when films are homogeneous and dense, differences in SEM cross-section images due to treatments are difficult to observe. It is well known that cracks and pores can affect the barrier properties of films [1]. 

Compared to the film thermally treated for 30 min without CA, the surface roughness slightly increased when 2.5% CA—30 min was used. This increase in roughness could be attributed to the introduction of a new component in the matrix. Although roughness can affect the hydrophobicity of the surface of films, a higher content of CA and longer reaction times decreased roughness to some extent, probably due to an increase in the compatibility between all components. 

Films treated with citric acid showed larger number of white-colored spots on the surface, which could be ascribed to the presence of residual salts or PEC aggregation. Several authors observed PEC aggregation on the surface of chitosan-based films incorporated with different biobased extracts [50,51,52]. Energy-Dispersive Spectroscopy (EDS) analysis was conducted on the aggregates and on the matrix of films (7.5 wt% CA, 155 °C, 30 and 60 min). It was found that certain aggregates had a similar composition to that of the matrix (mainly C, O, N), and others showed the presence of salts (low concentrations of Ca, Mg, Na, S).

#### 3.5.4. Surface Wettability 

**Figure 7:**
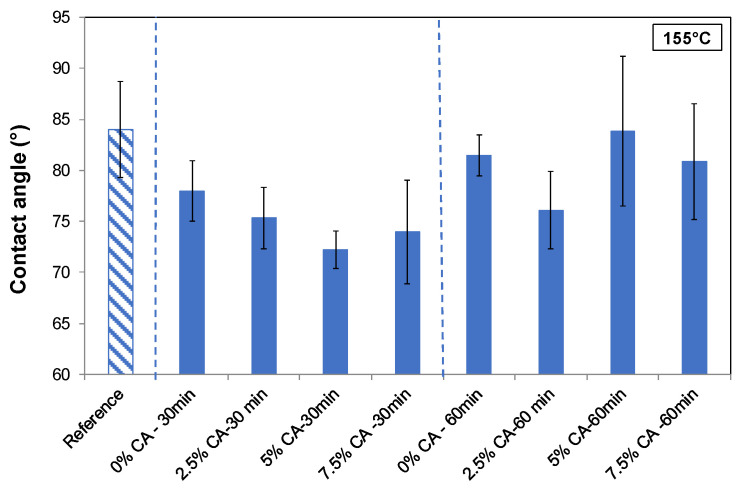
Contact angle results of the reference film (striped pattern), as well as thermally treated films (155 °C) for two times (30 min, 60 min) at different concentrations of CA (0 wt%, 2.5 wt%, 5 wt%, 7.5 wt% CA). Blue dashed lines separate different treatment times.

Figure 7 shows the contact angles of all the films. This property allows estimating the ability of the material to repel or absorb water, which is important for food packaging. A high water-contact angle indicates hydrophobicity (>90°), while a low contact angle indicates hydrophilicity (<90°). The figure shows that films of Xyl/Ch without treatment had a contact angle of 84°. The thermal treatment for 30 or 60 min did not markedly modify this value. Nevertheless, citric acid produced a decreasing tendency in this property compared to the thermally treated film, suggesting higher surface hydrophilicity. This behavior could be attributed to the mono and di-esters anchored to the carbohydrates, which left hydroxyl and carboxylic acid groups exposed to the medium. 

Ryan and Poduska [53] reported that a rough surface can reduce the contact angle for a droplet when the surface is hydrophilic, in agreement with the results of our work. Taking into account SEM results (Figure S3), rougher films (2.5% CA—30 min; 7.5% CA—30 min) showed lower contact angles. In contrast, films thermally treated for a longer time (60 min) containing citric acid showed both smoother surfaces and higher contact angles, reaching the values of the reference film and confirming acceptable compatibility between the components in the matrix. 

All obtained values indicate that films are slightly hydrophilic. They also indicate that films are of the same order of hydrophilicity or higher than other polysaccharide-based films reported in the literature. For example, Wu et al. [40] obtained potato starch/chitosan composite films by crosslinking with citric acid (5 to 20% at 55° C) whose contact angles were between 37° and 56°. Gordobil et al. [2] prepared xylan and acetylated xylan films containing different concentrations of cellulose or acetylated cellulose, respectively, observing values ranging from 57° (xylan film) to 77° (acetylated xylan/acetylated cellulose film). On the other hand, using chitosan/acorn starch/eugenol films, Zheng et al. [4] obtained values ranging from 74°, for chitosan/acorn starch films, to 89° when there was eugenol. 

Wu et al. [40] and Jiang et al. [54] reported that the hydrophobicity of biobased films depends on the structure of the network, which can be improved by crosslinking. They also used citric acid, suggesting that this phenomenon may be linked to a reduction in polar groups due to the formation of ester groups between citric acid and polymers. Wu et al. [40] found that, when a high citric acid concentration was used (20%), the contact angle decreased, probably due to the free hydrophilic groups (–COOH and –OH) from the excessive addition of citric acid. 

#### 3.5.5. Thermal Stability

Figure S4 shows the thermogravimetric curves of the different films; Table 2 summarizes characteristic temperatures (T_onset_, T_deg_, and T_endset_), and the percentage of mass residue at 800 °C (W_residual_). The figure shows a first thermal event in which mass loss was seen in all films. This event occurred in a temperature range of 54–110 °C and can be attributed to the loss of absorbed moisture from the films. It was found that, in this temperature range, the reference film lost 11% of the total mass, while for thermally treated films (30 or 60 min), a minor mass loss of around 7.5% was found, indicating that films absorbed less water. However, although thermal treatment was applied, the presence of citric acid in the films increased the percentage of water mass absorbed to 10–15%, probably because the mono- and di-esters anchored to the carbohydrates left hydrophilic groups exposed to the medium. 

Table 2 shows that the thermal properties of the reference film were modified after the different treatments. The lowest T_onset_ value was obtained for the reference film (224 °C). For the rest of the treatments, except for the film treated at 155 °C for 30 min, this value increased up to around 230 °C, indicating higher thermal stability. This increase is expected, as more crosslinks were formed within the network. In general, T_deg_ was also increased by the treatments. 

The solid residue of the reference film at 800 °C was around 5%, while the solid residue for films treated at 155 °C, regardless of whether citric acid was present, was around 10–31%. This increase also indicates that thermal resistance was markedly improved. Guevara et al. [55] reported a similar behavior when chitosan/pectin films were crosslinked with calcium chloride, thus concluding that the increase in thermal stability when the crosslinking agent was present was attributed to a more complex structure that delayed thermal decomposition. 

## 4. Conclusions

Highly transparent and homogeneous films with good UV-blocking performance were prepared from xylan and chitosan stable polyelectrolyte complex suspensions containing different concentrations of citric acid (0 wt%, 2.5 wt%, 5 wt%, 7.5 wt% CA). They also exhibited smooth, continuous, and partly wettable surfaces. 

Amidation and esterification reactions between polysaccharides and a citric acid crosslinking agent substantially contribute to the enhancement of the dry and wet mechanical properties of films, as well as their water resistance. Major favorable changes were observed when thermal treatment was applied at 155 °C. However, the citric acid slightly improved these properties mainly when 2.5 wt% CA and 5 wt% CA were used. The reactions also produced mono- and di-esters anchored to the carbohydrates, which could limit improvement in the properties, mainly the water resistance of the films. The results also indicate that crosslinking reactions and carbohydrate hydrolysis are concomitant processes that depend on temperature and citric acid concentration. 

The thermal stability of the films increased due to thermal treatments in the presence of citric acid, reaching an onset temperature of 230 °C for all films. 

These results suggest that biobased films with interesting mechanical properties can be obtained, contributing to environmental protection and preservation.

## Figures and Tables

**Table 1:** Characterization of Xyl/Ch PEC suspensions in the presence of different concentrations of citric acid and catalyzer.

Identification	Z-Potential (mV)	Average Particle Size (nm)	PDI	meq(+)/g PEC
0% CA	37.15 ± 0.46	1078 ± 24	0.271 ± 0.030	0.99
2.5% CA	35.52 ± 0.76	1037 ± 27	0.286 ± 0.030	0.83
5% CA	34.11 ± 0.53	921 ± 27	0.217 ± 0.030	0.68
7.5% CA	30.05 ± 0.26	876 ± 17	0.239 ± 0.040	0.54

**Table 2:** Characteristic temperatures.

Identification	T_onset_ (°C)	T_deg_ (°C)	T_endset_ (°C)	W_residual_ (%)
Reference	224	246	280	4.6
0% CA—30 min	224	247	293	28.4
2.5% CA—30 min	229	250	285	11.0
5% CA—30 min	230	254	289	27.2
7.5% CA—30 min	230	250	280	13.9
0% CA—60 min	231	255	295	29.8
2.5% CA—60 min	231	255	302	31.0
5% CA—60 min	229	246	289	31.7
7.5% CA—60 min	231	252	282	9.7

## Data Availability

The original contributions presented in the study are included in the article/Supplementary Materials, further inquiries can be directed to the corresponding author.

## Supplementary Materials

The following supporting information can be downloaded at https://www.mdpi.com/article/10.3390/polym16172407/s1, Figure S1: FTIR spectrum of the citric acid powder used in this work; Figure S2: Confocal Raman spectrum of the Xyl/Ch reference film (the region between 1800 and 1200 cm^−1^ is explained in detail in Figure 2); Figure S3: Surface Scanning Electron Micrographs of 70 Xyl/30 Ch wt% films prepared using different concentrations of citric acid (0, 2.5, 5, and 7.5 wt%) and thermally treated at 155 °C for different times (30 and 60 min). The magnification was 8000×; Figure S4. Thermogravimetric curves of the different films; Table S1: Opacity values of the films calculated using Equation (1) at 660 nm.
