# Supplementary material for: Tuning the Properties of Xylan/Chitosan-Based Films by Temperature and Citric Acid Crosslinking Agent
Source: Polymers (Basel). 2024 Aug 24;16(17):2407. doi: 10.3390/polym16172407 (PMC11397609; doi:10.3390/polym16172407)
Supplement: Supplementary file 1 [file polymers-16-02407-s001.zip › polymers-3152653-supplementary.pdf]

Supplementary material

TUNING THE PROPERTIES OF XYLAN/CHITOSAN-BASED FILMS BY TEMPERATURE AND CITRIC ACID CROSSLINK

Martina Camaño Erhardt [1], Yamil Nahún Solier [1,2], María Cristina Inalbon[1,2], Paulina Mocchiutti[1,2,*]

[1] Instituto de Tecnología Celulósica, Facultad de Ingeniería Química, Universidad Nacional del Litoral, Santiago del Estero 2654, S3000AOJ, Santa Fe, Argentina. martinacamano2@gmail.com (M.C.E); ysolier@fiq.unl.edu.ar (Y.N.S); cinalbon@fiq.unl.edu.ar (M.C.I); paulinam@fiq.unl.edu.ar (P.M)
[2]Consejo Nacional de Investigaciones Científicas y Técnicas (CONICET), Argentina.

[*]Correspondence: paulinam@fiq.unl.edu.ar; +54 342 4571160

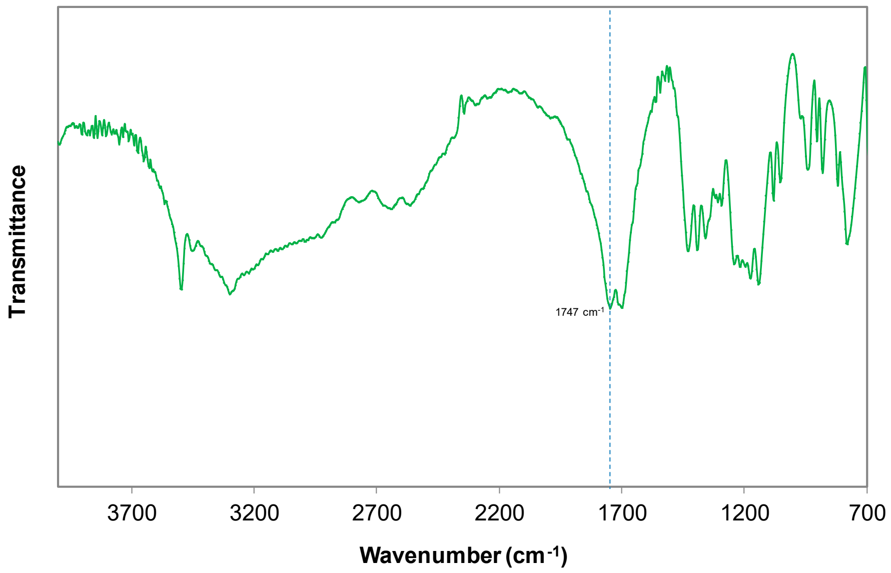

Fig. S1. FTIR spectrum of the citric acid power used in this work.

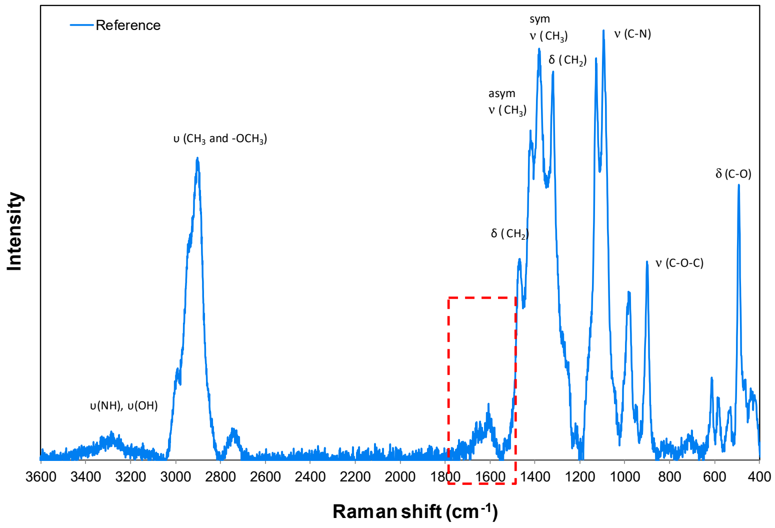

Fig. S2. Confocal Raman spectrum of the Xyl/Ch reference film (the region between 1800 cm^{-1} and 1200 cm^{-1} is explained in details in Figure 2).

Table S1. Opacity values of the films calculated using equation (1) at 660 nm.

	**Opacity (660 nm)**
Reference	3.5
0% CA - 30 min	2.2
2.5% CA - 30 min	1.9
7.5% CA - 30 min	0.9
0% CA - 60 min	1.6
2.5% CA - 60 min	1.8
7.5% CA - 60min	2.4

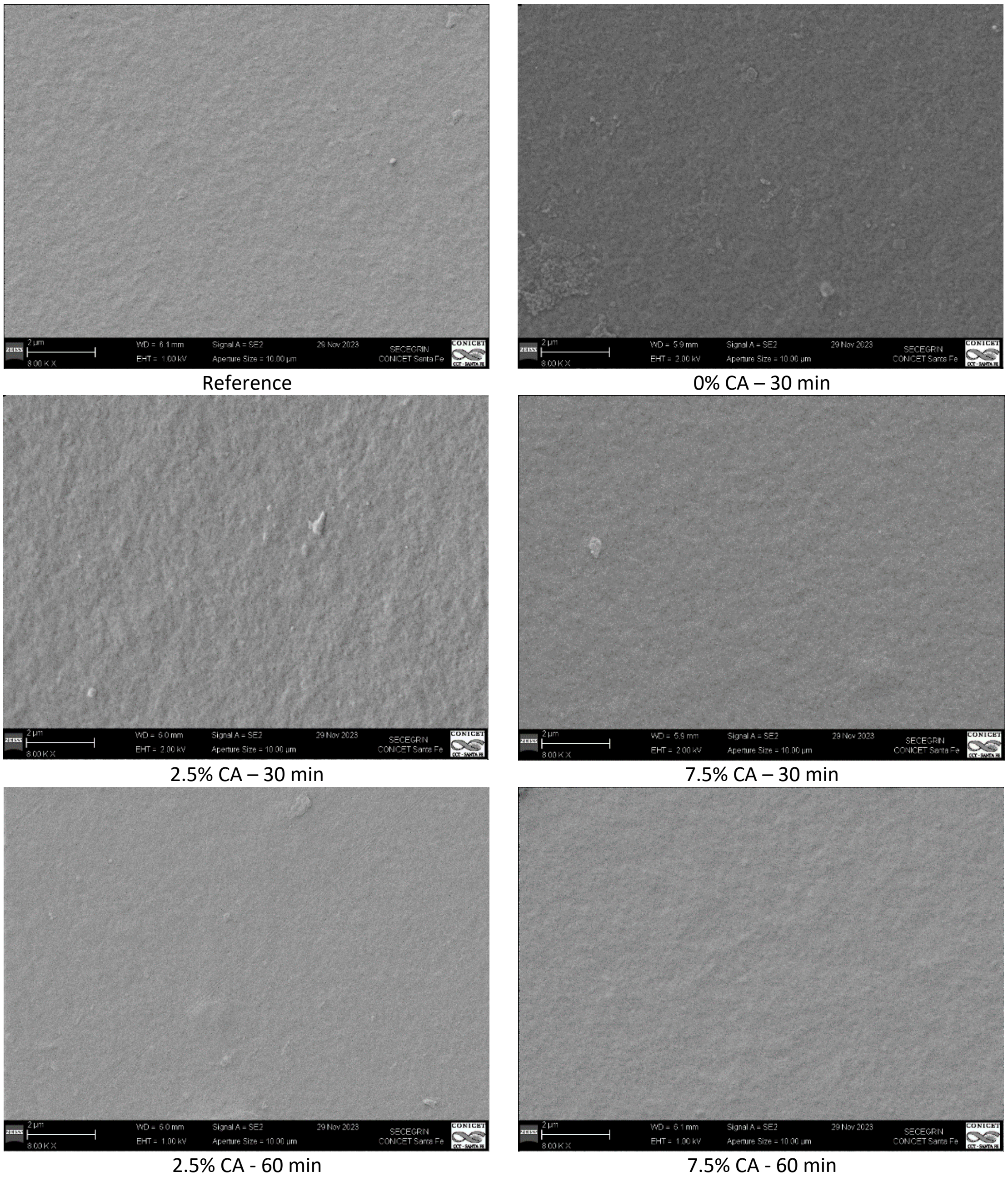

Fig.S3. Surface Scanning Electron Micrographs of 70 Xyl/30 Ch wt.% films prepared using different concentrations of citric acid (0, 2.5, 5 and 7.5 wt.%) and thermally treated at 155°C for different times (30 min and 60 min). The magnification was 8000 X.

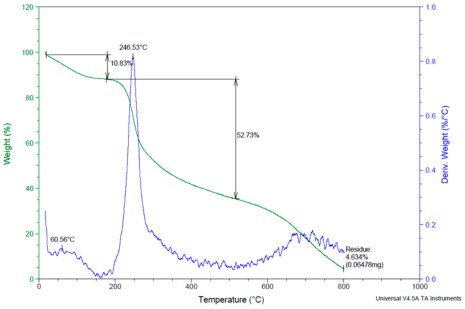

Reference

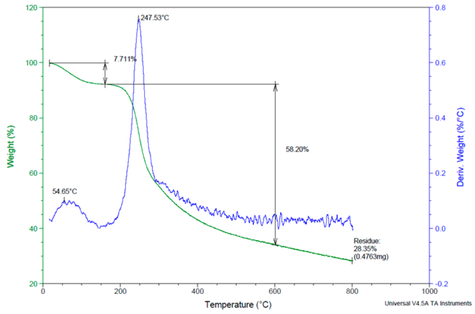

0% CA - 30 min

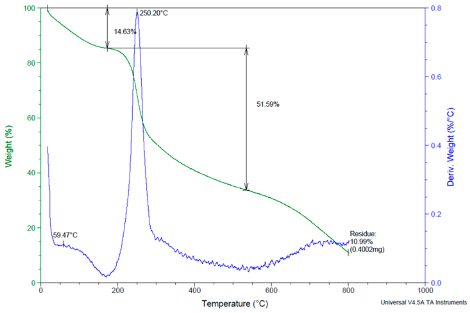

2.5% CA - 30 min

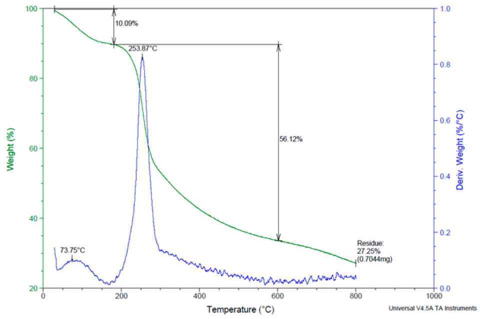

5% CA - 30 min

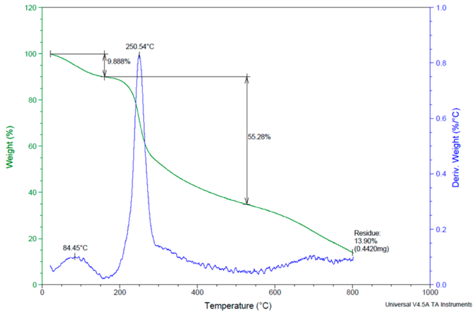

7.5% CA - 30 min

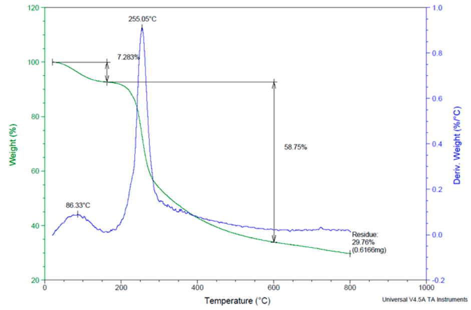

0% CA - 60 min

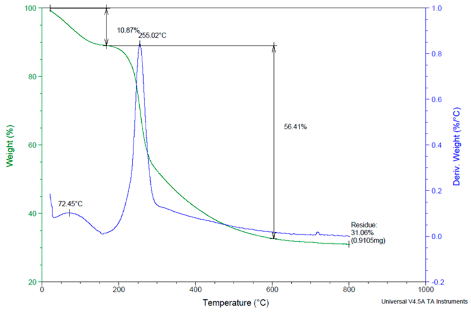

2.5% CA - 60 min

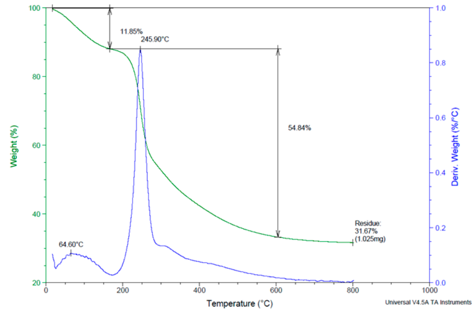

5% CA – 60 min

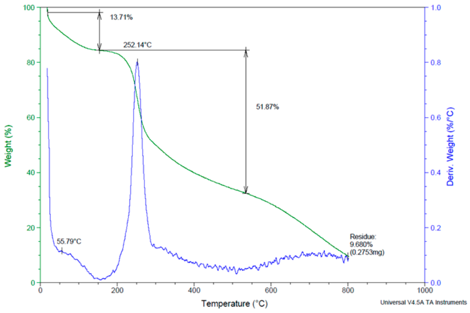

7.5 % CA – 60 min

Fig.S4. Thermogravimetric curves of the different films
